# Ameliorative Effects of Cumin Extract-Encapsulated Chitosan Nanoparticles on Skeletal Muscle Atrophy and Grip Strength in Streptozotocin-Induced Diabetic Rats

**DOI:** 10.3390/antiox13010006

**Published:** 2023-12-19

**Authors:** Yu-Chiuan Wu, Min-Chien Su, Chun-Shien Wu, Pin-Yu Chen, I-Fen Chen, Feng-Huei Lin, Shyh-Ming Kuo

**Affiliations:** 1 Republic of China Military Academy, Kaohsiung 830208, Taiwan; ranger.wu1113@gmail.com; 2 Kaohsiung Armed Forces General Hospital, Kaohsiung 802301, Taiwan; 3 Department of Biomedical Engineering, I-Shou University, Kaohsiung 84001, Taiwan; michelle.su1510@gmail.com (M.-C.S.); christina64270518@gmail.com (P.-Y.C.); ifen@isu.edu.tw (I.-F.C.); 4 Center of General Education, I-Shou University, Kaohsiung 84001, Taiwan; wucs@isu.edu.tw; 5 Department of Biomedical Engineering, National Taiwan University, Taipei 10617, Taiwan; double@ntu.edu.tw

**Keywords:** cumin, muscle atrophy, chitosan nanoparticles, antioxidant, diabetic

## Abstract

Skeletal muscle atrophy is a disorder characterized by reductions in muscle size and strength. Cumin extract (CE) possesses anti-inflammatory, antioxidant, and hypoglycemic properties. Its pharmaceutical applications are hindered by its low water solubility and by its cytotoxicity when administered at high doses. In this study, we have developed a simplified water distillation method using a rotary evaporator to isolate the active components in cumin seeds. The anti-inflammatory effects of CE and its potential to ameliorate skeletal muscle atrophy in rats with streptozotocin (STZ)-induced diabetes were evaluated. The half-maximal inhibitory concentration (IC_50_) of CE for cells was 80 μM. By encapsulating CE in chitosan nanoparticles (CECNs), an encapsulation efficacy of 87.1% was achieved with a slow release of 90% of CE after 24 h of culturing, resulting in CECNs with significantly reduced cytotoxicity (IC_50_, 1.2 mM). Both CE and CECNs significantly reduced the inflammatory response in interleukin (IL)-6 and IL-1β assays. STZ-induced diabetic rats exhibited sustained high blood glucose levels (>16.5 mmol/L), small and damaged pancreatic β islets, and skeletal muscle atrophy. CE and CECN treatments ameliorated skeletal muscle atrophy, recovered muscle fiber striated appearance, increased grip strength, and decreased IL-6 level. Furthermore, CE and CECNs led to a reduction of damage to the pancreas, restoring its morphological phenotype, increasing serum insulin levels, and lowering blood glucose levels in STZ-induced diabetic rats. Taken together, treatment with CECNs over a 6-week period yielded positive ameliorative effects in STZ-induced rats of muscle atrophy.

## 1. Introduction

Skeletal muscle atrophy is a common complication associated with both aging and a number of health problems, including chronic obstructive pulmonary disease, chronic heart failure, cancer, and inflammation. The underlying mechanisms of skeletal muscle atrophy involve the upregulation of specific proteins and inflammation through the NF-κB signaling pathway, which indirectly or directly disrupts muscle regeneration, thereby leading to skeletal muscle atrophy [1]. Although skeletal muscle atrophy is not life threatening, it can contribute to the development of complications such as osteoporosis, thrombosis, and pressure ulcers, all of which can indirectly increase morbidity and mortality rates.

Currently, the most common treatment methods for skeletal muscle atrophy are physical rehabilitation therapy and drug therapy. Drug therapy involves the use of medications such as anabolic drugs, enzyme inhibitors, and anti-inflammatory drugs, including androgen/androgen receptor modulators, ghrelin, β-adrenoceptor agonists, Cox2 inhibitors, histone deacetylase inhibitors, and phosphodiesterase inhibitors [2]. However, some of these drugs can have side effects, such as prostatitis, blood embolism, gastrointestinal complications, dizziness, and chronic diseases. Therefore, exploring the potential of natural compounds for the treatment of skeletal muscle atrophy may be a promising approach. Numerous studies have demonstrated that natural compounds can prevent or treat skeletal muscle atrophy. Examples of such natural compounds are resveratrol, salidroside, imperatorin, and ursolic acid [2].

The seed of the cumin plant is a widely used spice worldwide and constitutes the main ingredient in curry powder. The major volatile substances found in cumin seeds are terpenoids, cymene, and cumin aldehyde [3]. Cumin seeds also contain flavonoids, which are known for their antioxidant activity [4]. Miah et al. indicated that cumin seed powder supplementation in rats fed with a high-fat diet markedly suppressed oxidative stress in both their plasma and livers [5]. Cumin oil, which can be extracted from cumin seeds, has been used as an ingredient in some cosmetic products [6]. Cumin aldehyde, another extract from cumin seed, has demonstrated the ability to scavenge the superoxide anion [7]. Cumin oil has been shown to possess anti-inflammatory properties and has been found to inhibit the mRNA expression of interleukin (IL)-1β and IL-6, which are inflammatory factors, in lipopolysaccharide (LPS)-induced inflamed RAW264.7 macrophages. This effect occurs through the modulation of the JNK, ERK, and NF-κB signaling pathways [8]. Langen demonstrated that high levels of tumor necrosis factor (TNF)-α and IL-1β inhibited the differentiation of cultured myoblasts into myotubes through the activation of the NF-κB transcription factor [9]. This was also found to have cardioprotective, hypotensive, and lipid-lowering activities [5,10]. In addition, cumin exerts a hypoglycemic effect on streptozotocin induced diabetic rats [11,12]. However, the low water solubility, volatility, and cytotoxicity of CE impede its potential pharmaceutical applications, particularly for treating chronic diseases such as diabetes.

Diabetes is a prevalent chronic disease that affects a large proportion of the global population. Physiologically, insulin plays a crucial role in regulating skeletal muscle protein via the AKT/mTOR pathway. In health conditions where blood glucose levels are high and where insulin levels are low, protein synthesis is inhibited and protein breakdown is promoted. This imbalance leads to skeletal muscle atrophy and rapid weight loss [13]. Moreover, diabetes can force the immune system to secrete cytokines, which trigger various inflammatory responses. Prolonged chronic inflammation has been shown to increase the occurrence of catabolic diseases. Among the inflammatory cytokines, TNF-α is known to trigger excessive inflammation in the body, leading to skeletal muscle atrophy [14]. Skeletal muscle atrophy is often characterized by a decrease in the cross-sectional area of muscle fiber, an increase in protein catabolism, and subsequent loss of muscle mass and strength [15]. Skeletal muscle atrophy can be due to either increased protein degradation or decreased protein synthesis. Sustained inflammation, high levels of oxidative stress, and upregulated levels of pro-inflammatory cytokines such as TNF-α and IL-1β contribute to the degeneration of healthy muscle cells, resulting in skeletal muscle atrophy [16]. Furthermore, the elevation of inflammatory factors such as TNF-α, IL-1β, and IL-6 has been observed in human and chronic disease animal models of skeletal muscle atrophy [17,18].

In the treatment of diseases with drugs, the location, timing, and profile of drug release must be controlled to minimize toxicity and increase the efficacy of the drug. Nanoparticle encapsulation of drugs has emerged as a promising approach in the field of medicine [19]. Nanoparticles, with sizes ranging from 1 to 100 nm, offer advantages such as high drug loading capacity and specific targeting of cells owing to their size-dependent effect and intracellular uptake [20]. Biopolymeric nanoparticles—including nanosized hyaluronan, chitosan, and gelatin—are particularly favored as carriers in drug delivery systems because of their biocompatible and biodegradable properties [21]. In our previous studies, we have demonstrated that encapsulating heteronemin by using chitosan and hyaluronan nanoparticles can enhance the antitumor effects of heteronemin [22]. Chitosan, a natural cationic polymer, has demonstrated properties such as absorption enhancement through mucosa, controlled drug release, and bioadhesion [23,24]. By considering the presence of the substantial amounts of beneficial reports of cumin and the drug carrier using chitosan, the current study was designed to investigate the anti-inflammatory effects of CE and CE-encapsulated chitosan nanoparticles (CECNs, which were fabricated using the electrostatic field system method) on C2C12 cells and the ameliorative effect on skeletal muscle atrophy in STZ-induced diabetic rats.

## 2. Materials and Methods

The dried cumin used in this study was purchased from a local store and was authenticated by Prof. Dr. Lin Li-wei. A voucher sample with the identification number ISU-MCMM-201 was preserved for future reference in the School of Chinese Medicines for Post-Baccalaureate, I-Shou University. STZ was purchased from Sigma (St. Louis, MO, USA), and 3-4,5-Dimethylthiazol-2-yl-2,5-diphenyltetrazolium bromide (MTT), Dulbecco’s Modified Eagle Medium, fetal bovine serum, streptomycin, and penicillin were obtained from Gibco (Waltham, MA, USA). All chemicals used in the present study were of reagent grade. Animal experiments conducted in this study were approved by the Institutional Animal Care and Use Committee of I-Shou University, Kaohsiung, Taiwan (IACUC-ISU-110–052, approval date: 4 March 2022).

### 2.1. Isolation and Characterization of CE

**Figure 1:**
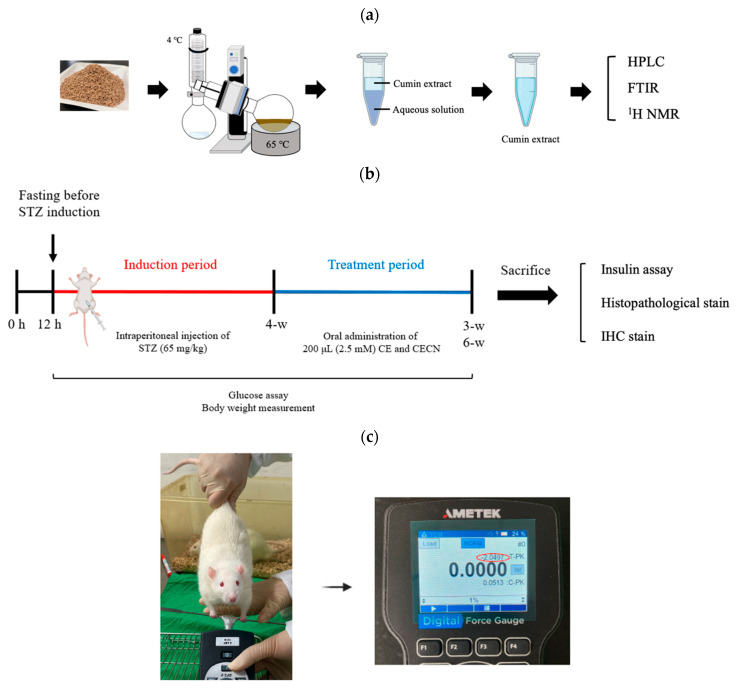
Schematic of (**a**) the procedure for the extraction of cumin seeds, (**b**) the timeline and experimental design for in vivo animal studies, and (**c**) grip strength measurement.

CE was extracted using the water distillation method. This procedure involved grinding 200 g of cumin seeds into a powder and then placing this powder in a rotary bottle. To this powder, 600 mL of ddH_2_O was then added. The extraction was conducted using a rotary evaporator (EYELA 1300VF, Tokyo, Japan) under the following conditions: vacuum pressure, 200 hPa; rotary speed, 20 rpm; heating temperature, 65 °C; condensation temperature, 4 °C; and extraction period, 3 h. The obtained CE was further treated with anhydrous sodium sulfate to remove residual water, and the resultant extract was stored at 4 °C for further experiments. Fourier-transform infrared (FTIR) spectroscopy and ^1^H nuclear magnetic resonance (NMR) analyses were performed to identify the CE, as compared with the pure cumin purchased from Sigma. FTIR spectroscopy was employed to characterize the characteristic peaks of CE (Figure 1a). The yield of extraction was determined using the following formula:

Yield of extraction (YE, %) = (weight of CE/weight of cumin seeds) × 100%

High-performance liquid chromatography (HPLC, Agilent 1100, Santa Clara, CA, USA) equipped with a Zoxbax-C18 column was employed to determine the chemical composition of the CE. The mobile phase consisted of a mixture of sodium sulfate, acetonitrile, and methanol (90:190:20, *v*/*v*), and the flow rate was set at 1 mL/min. Detection was performed at 326 nm using an ultraviolet–visible detector. The characteristic peaks of CE were further analyzed through FTIR spectroscopy with potassium bromide disks (Jasco 4700, Tokyo, Japan), and the NMR spectra were recorded using a Bruker AM-400 spectrometer (Bruker Avance III HD 600 MHz, Karlsruhe, Germany).

### 2.2. Fabrication of CECNs

Chitosan nanoparticles were prepared by following the methodology described in a previous study [25]. In brief, an EFS was established using two parallel copper plates (18 cm × 6 cm) spaced 2 cm apart. The electrical field strength between the plates was generated using a direct-current power supply. The EFS was constructed in a thermal chamber for temperature control and safety purposes. To prepare the CECNs, 1 mL of chitosan solution (0.2 mg/mL) was carefully mixed with 150 μL of CE (0.5 M). This mixture was then transferred onto a petri dish and placed between the two plate electrodes in the EFS. The following conditions were set for CECNs fabrication in the EFS: temperature, 17 °C; applied electrical field strength, 2.5 kV/cm; reaction time, 5 h; and crosslinking reagent, 0.001 N NaOH. The sizes of the chitosan nanoparticles and CECNs were determined by randomly sampling approximately 60 individual nanoparticles through transmission electron microscopy (TEM). To assess the entrapment efficiency (EE) of CE, 1 mL of the solution containing the prepared CECNs was centrifuged at 10,000 rpm for 10 min. The amount of nonencapsulated CE in the supernatant was measured through HPLC. A standard concentration curve of CE was established to determine the amount of CE encapsulated in CECNs. Finally, the EE was calculated using the following formula: [(total amount of CE − amount of nonencapsulated CE)/total amount of CE] × 100%. Next, the in vitro release of CE from CECNs was evaluated by adding 100 μL of CECNs to a 1.5 mL microcentrifuge tube, which was then placed in a shaker set at 37 °C and 40 rpm. At predetermined time points, the sample was centrifuged at 10,000 rpm for 10 min. The amount of nonencapsulated CE in the supernatant was determined through HPLC. Independent experiments were performed three times in triplicate. The in vitro release rate was calculated as follows: [(total amount of CE − amount of residual CE)/total amount of CE] × 100%.

To determine the volatility of CE and CECNs, 1 mL of CE or CECNs solution was added to a 48-well plate, and this plate was placed in a 37 °C, 5% CO_2_ incubator. At predetermined time points, the sample was centrifuged at 10,000 rpm for 10 min. The amount of residual CE in the supernatant was then determined through HPLC. The volatility of CE or CECNs was calculated as follows: 1 − [(total amount of CE − amount of residual CE)/total amount of CE] × 100%.

### 2.3. Cytotoxicity of CE and CECNs in Relation to C2C12 Cells

To assess the cytotoxicity of CE and CECNs in relation to C2C12 cells (ATCC, VA, USA), the MTT assay was performed. The C2C12 cells were seeded on a 96-well plate at a density of 1.2 × 10^4^ cells/well and were allowed to attach for 24 h. Subsequently, these cells were treated with multiple concentrations of CE (20–100 μM) and CECNs (0.6–1.4 mM). After 24 h of treatment, 10 μL of MTT solution (5 mg/mL) was added to each well, and the plate was then incubated for an additional 4 h. To dissolve the formazan precipitate, DMSO was added to each well. The absorbance of the formazan solution was measured at 450 nm using a multiplate reader (Thermo Scientific, Waltham, MA, USA).

### 2.4. Evaluation of the Anti-Inflammatory Effects of CE and CECNs on LPS-Induced C2C12 Cells

To evaluate the anti-inflammatory effects of CE and CECNs on LPS-induced C2C12 cells, C2C12 cells (1.2 × 10^4^ cells/well) were seeded on a 24-well plate for 24 h and preincubated with 0.3 mg/mL LPS for 6 h. These cells were then treated with multiple concentrations of CE (20, 30, and 40 μM) and CECNs (0.6, 0.7, and 0.8 mM). After incubation for 24 h, the medium was collected and centrifuged at 1000× *g* for 20 min to obtain cell-free supernatant, which was then used to perform the enzyme-linked immunosorbent assay (ELISA) for IL-6, IL-1β, and TNF-α. The levels of IL-6, IL-1β, and TNF-α were quantified using the Elabscience^®^ Mouse IL-6, IL-1β, and TNF-α ELISA Kit (Minneapolis, MN, USA), and absorbance was measured at 450 nm according to the manufacturer’s protocol.

### 2.5. Establishment of the STZ-Induced Diabetic Rat Model

Animal experiments were performed over 10 weeks (Figure 1b). Diabetes was induced in rats by using STZ. A 1% STZ working solution (Sigma-Aldrich, St. Louis, MO, USA) was prepared in 50 mmol citrated buffer solution (pH 4.2). A total of 10 male rats were used to establish the diabetic rat model, whereas two rats served as the healthy control group. The rats were intraperitoneally injected with 200 μL of STZ solution (65 mg/kg body weight). After the injection, each rat was left untreated for 4 weeks. During this 4-week period, the rats’ blood glucose levels were measured at p.m. 14:00 every 3 days using EasyTouch^®^ (Yuan Yu Bio-Tech Co., Ltd., Taoyuan, Taiwan). Blood glucose levels of >16.0 mmol/L were considered indicative of diabetes [26]. The rats were anesthetized using Zoletil^®^ (intraperitoneal administration of 40 mg/kg of tiletamine with 50 mg/kg of zolazepam,) and xylazine (10 mg/kg) and randomly allocated into the following groups (n = 2 per group): Group A: STZ-induced diabetic rats without treatment, serving as a negative control group; Group B: rats treated with 200 μL of CE (2.5 mM) every 2 days for 3 or 6 weeks; and Group C: rats treated with 200 μL of CECNs (2.5 mM) every 2 days for 3 or 6 weeks. At weeks 3 and 6 after treatment, one rat was randomly selected from each group and sacrificed. The rectus femoris muscle, liver, pancreas, kidney, and serum of the sacrificed rats were harvested for histopathological and insulin analyses.

### 2.6. Histopathological Analyses

At weeks 3 and 6 after treatment, the sacrificed rats’ rectus femoris muscle, liver, pancreas, and kidney tissues were harvested and fixed in 10% neutral-buffered formalin. The resulting samples were then dehydrated in graded ethanol solution, cleared in xylene, embedded in paraffin blocks, and cut into 5 μm thick sections. Histopathological examination of the tissue samples was performed through hematoxylin and eosin (H&E) staining.

### 2.7. Forelimb Grip Strength Measurement

As shown in Figure 1c, the grip strength of the rats was assessed using a standardized method, where each rat was lifted by the tail and encouraged to grasp a rigid metal bar attached to a digital force gauge (AMETEK^®^ Measurement & Calibration Technologies, Inc., Miami, FL, USA). Subsequently, the rat was gently pulled backward by the tail until it released the metal bar. The tension reading displayed on the digital force gauge just before the rat released the metal bar was recorded as the grip strength. This test was performed three consecutive times with a 3 min rest after each round. Each rat’s grip strength measurement is expressed as mean ± standard error in this paper.

### 2.8. Insulin Assay

To assess the function of the rat pancreas, a blood biochemical parameter, namely insulin, was assayed. Rat serum samples were isolated from whole blood sample and subjected to insulin biochemical analysis according to the insulin manufacturer’s instructions (Elabscience^®^ Insulin ELISA kit, Houston, TX, USA).

### 2.9. Immunohistochemistry (IHC) Analysis

For (IHC) analysis, the sections were deparaffinized and rehydrated using graded concentrations of ethanol and then deionized water. The sections were incubated in a hydrogen peroxide blocking solution for 10 min and then washed with PBS. The sections were subjected to heat treatment (at 95 °C) in 0.01 M sodium citrate buffer with Tween 20. Each section was incubated with ImmunoBlock (PBS, pH 7.6, with 0.5% bovine serum albumin) at room temperature for 20 min, followed by washing with PBS. Next, they were incubated with rabbit anti-mouse IL-6 (Novus Biologicals, LLC, Centennial, CO, USA) at 4 °C overnight and then with Mouse/Rabbit Probe HRP Labeling solution at 25 °C for 30 min. Finally, 3,3′-diaminobenzidine was applied for 10 min. The intensity of the brown color, indicating the presence of IL-6 was semi-quantified using ImageJ software (Version 1.50; National Institutes of Health, Bethesda, MD, USA).

### 2.10. Statistical Analysis

In this paper, all data are expressed as mean ± standard error of the mean. Differences among groups were analyzed using a one-way analysis of variance followed by Tukey’s multiple comparison test. A *p* value of <0.05 was considered statistically significant. All statistical analyses were performed using SPSS (version 20.0).

## 3. Results

### 3.1. Characterization of CE

**Figure 2:**
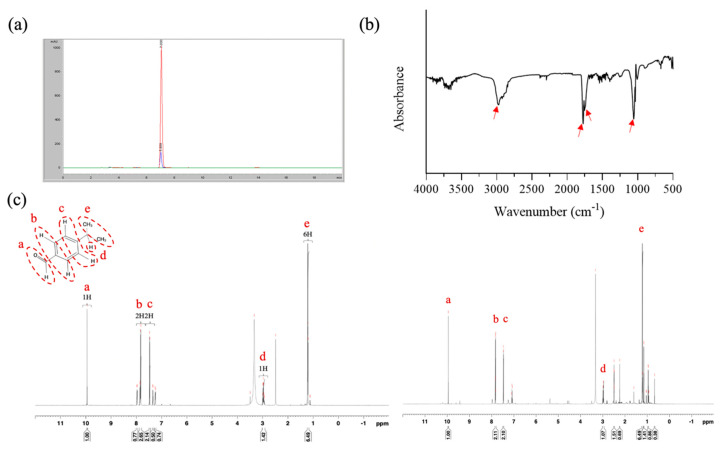
Characterization of CE. Results of (**a**) HPLC analysis (red color: pure cumin aldehyde; blue color: CE), (**b**) FTIR analysis, and (**c**) ^1^H NMR analysis (left panel: pure cumin aldehyde; right panel: CE).

The present study employed an easy extraction method involving a rotary evaporator to isolate CE; cumin aldehyde was used as a reference compound to confirm the composition of CE. HPLC analyses revealed that the retention times of pure cumin aldehyde and CE were 7.03 and 6.99 min, respectively, indicating that the main component of CE was cumin aldehyde (Figure 2a). The yield of the extraction process, determined using HPLC, was approximately 0.8%. FTIR performed on CE revealed absorption bands at approximately 2971, 1772, and 1770 cm^−1^, indicating the presence of an aldehyde group, a C=O stretch, and a C–C stretch, respectively, in the aromatic ring. Additionally, an absorption band at 1054 cm^−1^ suggested the presence of an alcohol group in the molecule. Accordingly, we concluded that our CE contained a small amount of cuminol likely because of the extraction method employed in this study (Figure 2b). To further confirm the composition, ^1^H NMR was performed on cumin aldehyde (600 MHZ, dDMSO), and the data revealed the following statistics: δ = 9.957 (1H, a), 7.782 (2H, b), 7.356 (2H, c), 3.015 (H, d), and 1.244 (6H, e) (Figure 2c). Notably, the NMR spectrum for CE exhibited peaks similar to those of pure cumin aldehyde, confirming that the main compound isolated from cumin was cumin aldehyde.

### 3.2. Characterization of CECNs

**Figure 3:**
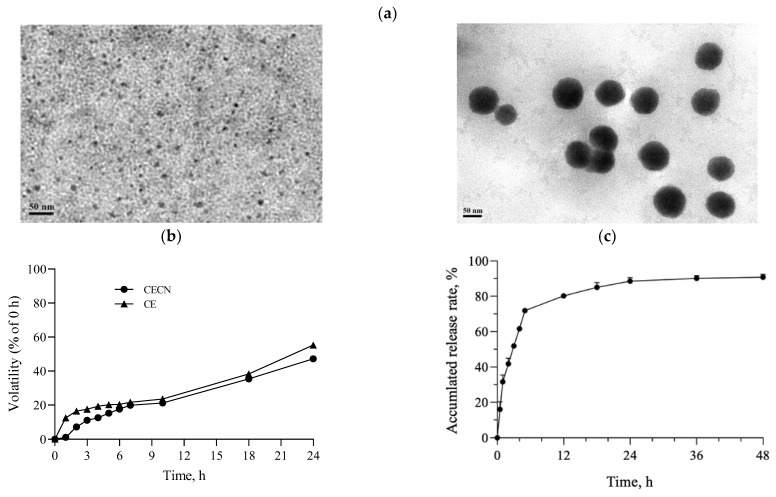
(**a**) TEM images of pure chitosan nanoparticles (left panel) and CECNs (right panel), (**b**) volatility profiles of CE and CECNs, and (**c**) release of CE from CECNs.

Figure 3a displays the TEM images of chitosan nanoparticles and CECNs prepared using an EFS in this study. The chitosan nanoparticles exhibited a well-dispersed and spherical morphology, with an average size of approximately 5.8 ± 1.3 nm. By contrast, the diameter of the CECNs was approximately 78.0 ± 14.8 nm, and the CECNs were formed through the aggregation of numerous chitosan nanoparticles and CE components. This aggregation can be attributed to interactions between the polar or nonpolar domains of chitosan and CE, leading to the formation of larger CECN structures. The volatility profiles of the CE and CECNs are presented in Figure 3b, which reveals the decreased volatility of the CECNs after encapsulation of CE within chitosan nanoparticles. The volatility of the CECNs was approximately 40% during a 24 h incubation period in a 37 °C, 5% CO_2_ incubator. However, the decrease in volatility after the encapsulation procedure was not substantial, possibly because of the encapsulation process, which involved the aggregation of numerous chitosan nanoparticles to trap the CE, leading to the formation of a loose and aggregated nanoparticle structure. The EE of the CE within the CECNs was approximately 87.3% ± 3.5%. The release pattern of CE from the CECNs was relatively rapid from 0 to 6 h; by the end of the 6 h period, approximately 72% of the encapsulated CE had been released from the CECNs, and after 48 h of incubation, approximately 90% of the encapsulated CE had been released from the CECNs.

### 3.3. Cytotoxicity and Anti-Inflammatory Effects of CE and CECNs on C2C12 Cells

**Figure 4:**
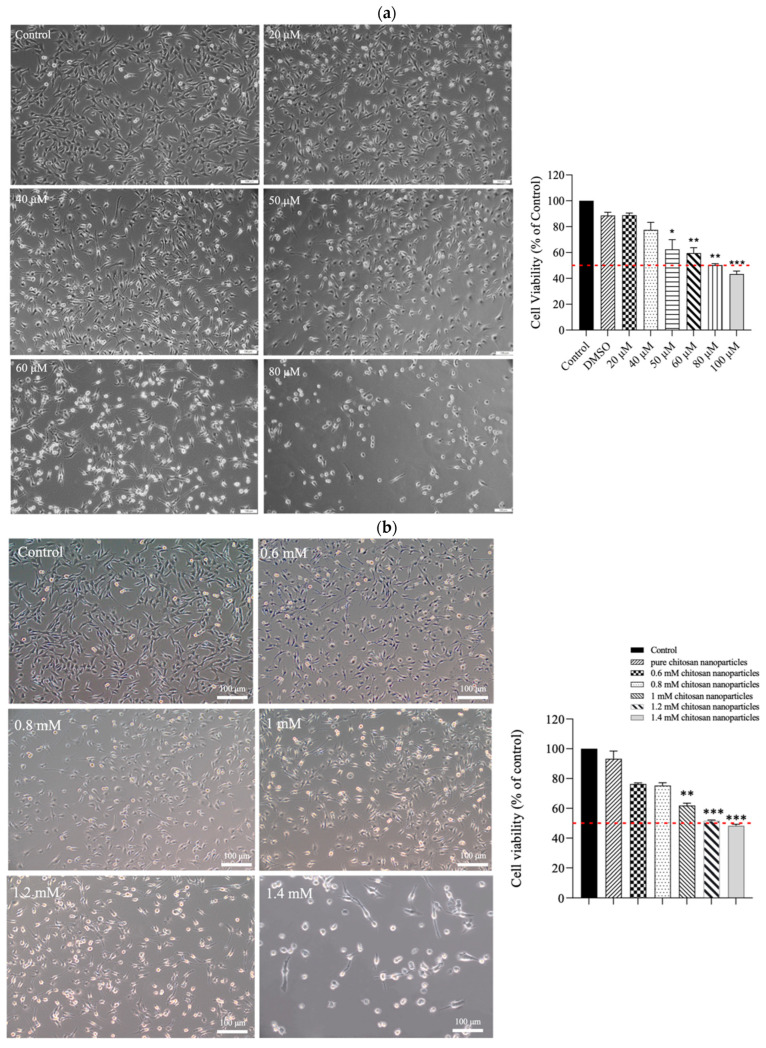

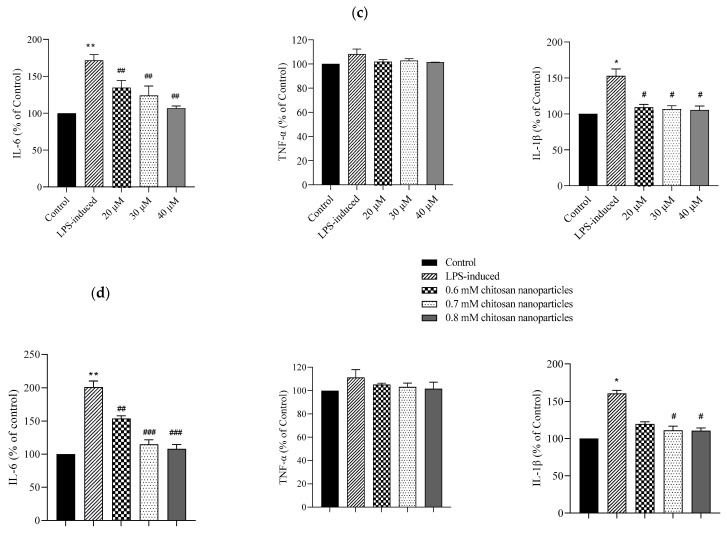
Cell morphologies and results of the MTT assay conducted on C2C12 cells treated with multiple concentrations of (**a**) CE and (**b**) CECNs. * *p* < 0.05, ** *p* < 0.01, and *** *p* < 0.001 compared with the control group. (**c**,**d**) Anti-inflammatory effects of LPS-induced C2C12 cells treated with multiple concentrations of CE and CECNs. * *p* < 0.05 and ** *p* < 0.01 compared with the control group. # *p* < 0.05, ## *p* < 0.01, and ### *p* < 0.001 compared with the LPS-induced group. Bar scale: 100 μm.

The results of the MTT assay revealed that CE exerted moderate cytotoxicity toward C2C12 cells. As depicted in Figure 4a, treatment with increasing concentrations (20–100 μM) of CE for 24 h led to a dose-dependent reduction in cell viability. The half-maximal inhibitory concentration (IC_50_) was approximately 80 μM. The morphological changes observed in the treated cells included the shrinkage of cell pseudopodia, the rounding of cells, and cell detachment from the culture surface, indicating that higher CE concentrations increasingly resulted in cell death. In contrast, when C2C12 cells were treated with CECNs, a considerable increase in cell viability was observed. The IC_50_ of the CECNs was approximately 1.2 mM, which was 15-fold higher than that of pure CE (Figure 4b). Similar to the CE treatment, the C2C12 treated with CECNs exhibited shrinkage, rounding, and detachment from the culture surface, indicating that higher CECNs concentrations increasingly resulted in cell death. This increase in the IC_50_ suggests benefits of the application of CE.

To induce inflammation in C2C12 cells, LPS was used. The minimum concentration of LPS required to trigger a major/severe inflammatory response in C2C12 cells was 0.3 mg/mL. Considering safety in relation to the C2C12 cells, we used half concentrations of the IC_50_ values of CE and CECNs to assess their anti-inflammatory effects. The anti-inflammatory effects of the CE and CECNs on C2C12 cells with LPS-induced inflammation are depicted in Figure 4c,d. Treatment with CE (20–40 μM) and CECNs (0.6–0.8 mM) effectively suppressed the levels of the inflammatory factors IL-6 and IL-1β. Additionally, the inflammatory factor TNF-α was slightly suppressed after treatment with the same concentration range of CE and CECNs.

Considering the cell cytotoxicity effect of CE and CECNs observed in the C2C12 cells and the oral administration route for treatment, the CE and CECNs concentrations were increased to 2.5 mM for the in vivo animal experiments conducted on STZ-induced diabetic rats.

### 3.4. Diabetes Induction and Histological Phenotyping

**Figure 5:**
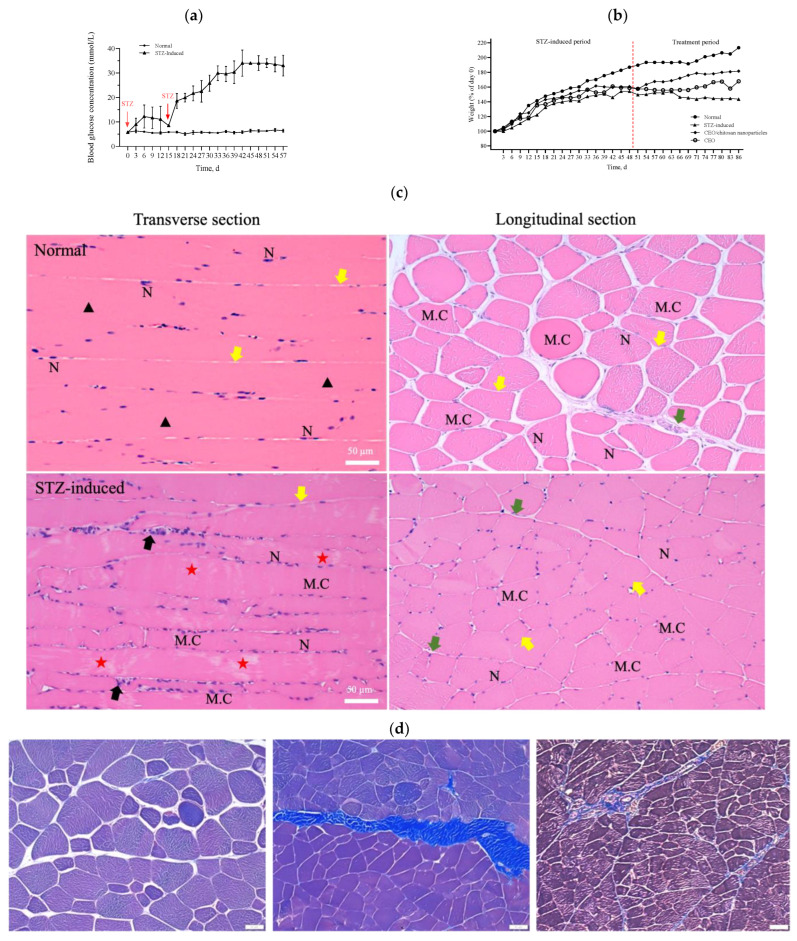

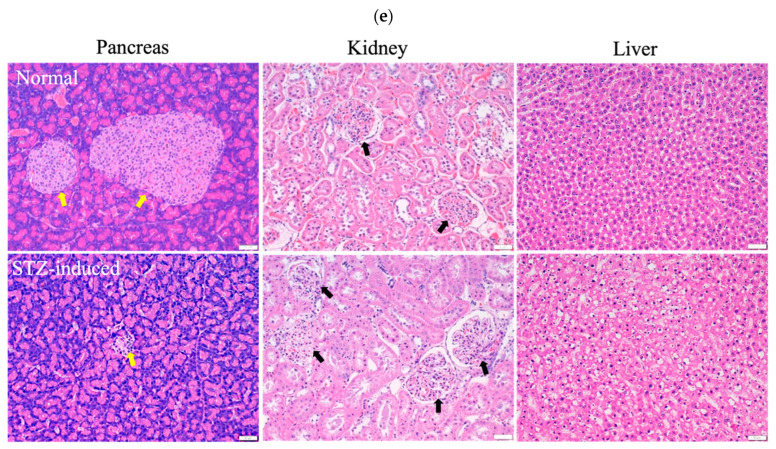
(**a**) Measurements of glucose levels. (**b**) Body weight changes during the in vivo experiment. (**c**) H&E staining of the rectus femoris muscle in normal and STZ-induced rats. ▲: regular transverse striations; ★: atypical muscle fibers, disrupted fibers with fewer striations. MC: muscle cell; N: nucleus; black arrow: inflammatory cell; green arrow: perimysium; yellow arrow: endomysium. (**d**) Masson trichrome staining of the rectus femoris muscle (left panel: normal group; right two panels: STZ-induced group). (**e**) H&E staining of pancreas, kidney, and liver samples in normal and STZ-induced rats. Yellow arrow: pancreatic islets of Langerhans, black arrow: kidney corpuscle. Bar scale: 50 μm.

During the experiment to induce diabetes in rats, the blood glucose levels of these rats did not increase to the value corresponding to the criteria for diabetes (i.e., >16 mmol/L) over 14 days after a single dose of STZ intraperitoneal injection. However, when an additional half dose of STZ was administered, the blood glucose levels markedly increased to values that met the criteria for diabetes (>20 mmol/L) and were significantly higher than the corresponding values of the healthy control group (Figure 5a). The body weight of the STZ-induced rats exhibited a mild and slow increase in the initial state followed by a progressive decline over a longer period (Figure 5b). In accordance with the results shown in Figure 5a,b, the STZ-induced diabetic rats were sacrificed 2 weeks after the second injection of STZ to ensure the development of diabetes in these rats. A histopathological examination of the rectus femoris muscle sections of normal rats revealed fibers with peripheral nuclei and acidophilic sarcoplasm (Figure 5c), regular transverse striations, a wide perimysium and endomysium area, and pale nuclei close to the sarcolemma. By contrast, sections from the STZ-induced diabetic rats revealed pale and uneven irregular cytoplasm texture, relatively few striations, disrupted fibers, aggregation of nuclei, inflammatory cells, and multiple macrophages in between muscle fibers (lower panel of Figure 5c: transverse section). Masson trichrome staining showed that collagen deposition was evident and significantly increased in the muscle tissue of diabetic rats compared with the normal group (Figure 5d). As shown in Figure 5e, H&E stains of pancreas, kidney, and liver samples revealed that the islets in the normal pancreas control rats exhibited a regular morphology with β cells located in the center. By contrast, in the STZ-induced diabetic rats, the pancreatic islets of Langerhans were smaller, had been destroyed, and contained many leukophilia leukocytes with some amyloid deposits. There were not any obvious morphological changes in the kidneys of the normal group or the STZ-induced group. In summary, the observed changes in blood glucose levels, body weight, morphology, collagen deposition in muscle tissue, and pathological changes in the pancreas indicated the successful establishment of a diabetic rat model.

### 3.5. Histological Results

**Figure 6:**
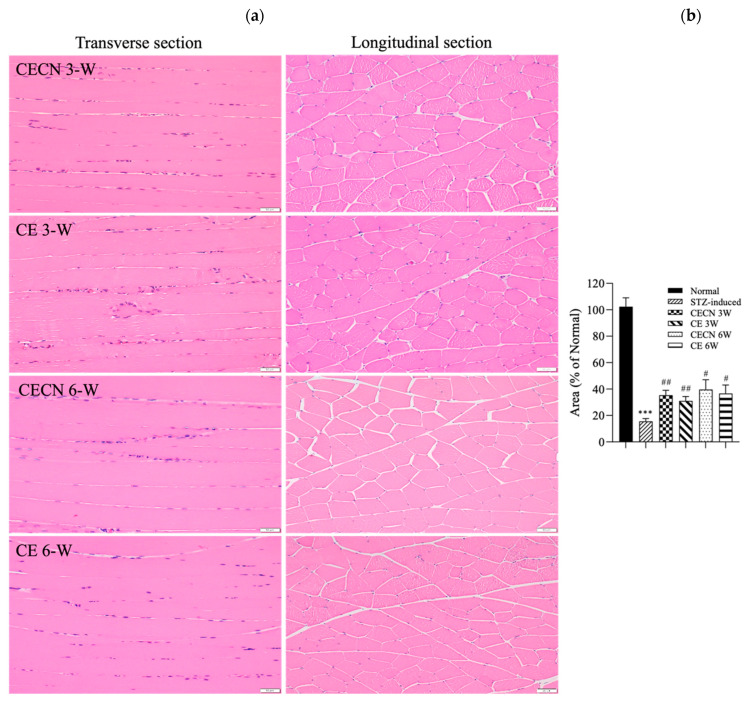
(**a**) H&E staining of the rectus femoris muscle after treatment with CE and CECNs for 3 and 6 weeks. (**b**) Semiquantitative determination of the area of the perimysium and endomysium from the longitudinal sections shown in (**a**) (three images). *** *p* < 0.001 compared with the normal group; # *p* < 0.05 and ## *p* < 0.01 compared with the STZ-induced group. Bar scale: 50 μm.

Cells in healthy skeletal muscle are divided longitudinally into numerous sarcomeres, which give skeletal muscle cells their striated appearance. Muscle physiology studies have suggested that the perimysium plays a role in transmitting lateral contractile movements [27]. Under normal circumstances, a gap exists between the muscle fibers, suggesting that the perimysium is related to the health and normal structure of the muscle. However, under conditions of muscle atrophy or muscle fiber degeneration, the spaces between muscle fibers become narrower, possibly because of a reduction in the number of muscle fibers or structural disturbances between these fibers. The narrowing of the perimysium and endomysium space observed in muscle tissue sections of the STZ-induced diabetic rats in this study may indicate muscle atrophy or the degeneration of muscle fibers. Accordingly, in STZ-induced diabetic rats, the narrowing of the perimysium and endomysium space may be a manifestation of muscle atrophy, suggesting that diabetes has an adverse effect on muscle tissue. This observation can be regarded as an indicator in the evaluation of the effect of therapeutic interventions on skeletal muscle atrophy. Histopathological analysis of H&E stains (Figure 6a) from the STZ-induced diabetic rats treated with CE or CECNs for 3 and 6 weeks revealed relatively pale nuclei close to the sarcolemma, a decreased number of inflammatory cells, and the restoration of the perimysium and endomysium space as compared with the STZ-induced groups (Figure 5c); however, some aggregation of nuclei was still observed. Notably, the muscle fibers exhibited the recovery of their striated appearance, and the area of the perimysium and endomysium was significantly enlarged compared with that of the STZ-induced rats (Figure 6b). Overall, the diabetic rats treated with CECNs and treated for the longer duration of 6 weeks exhibited a more favorable restoration effect on muscle morphology.

### 3.6. Grip Strength Measurement

**Figure 7:**
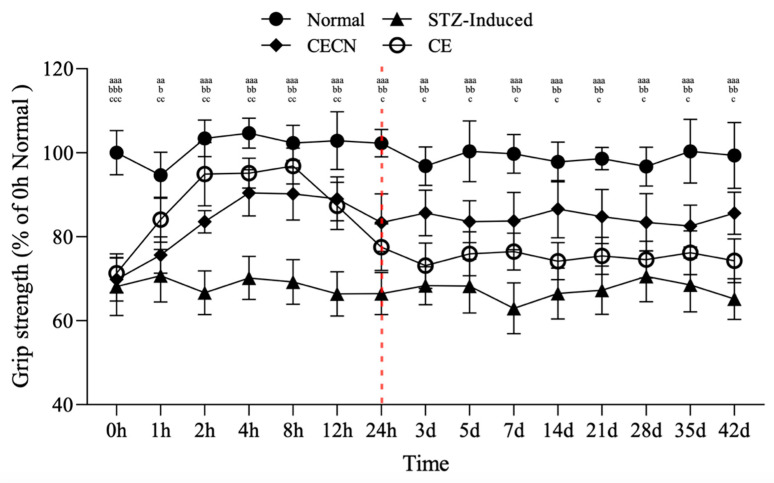
Changes in grip strength in STZ-induced diabetic rats after treatment with CE and CECNs. ^aa^ *p* < 0.001 and ^aaa^ *p* < 0.001 compared with the normal group; ^b^ *p* < 0.05, ^bb^ *p* < 0.01 and ^bbb^ *p* < 0.001 compared with the CECN group; ^c^ *p* < 0.05, ^cc^ *p* < 0.01 and ^ccc^ *p* < 0.001 compared with the CE group (n = 5 per group).

The forelimb grip strength test was conducted to assess the effects of CE and CECNs on skeletal muscle function in the STZ-induced diabetic rats over a 6-week period. This test employed the device shown in Figure 1c. In the STZ-induced group, grip strength significantly decreased to approximately 65% compared with that of the normal group (Figure 7). However, after oral administration of CE or CECNs, grip strength significantly increased compared with that of the STZ-induced group. In particular, CE exerted a more direct and rapid effect on grip strength, reaching approximately 94% of that in the normal group within the initial 8 h period. However, grip strength subsequently gradually decreased over time and became lower than that enabled by CECNs. Evidently, CECNs resulted in more favorable recovery of grip strength compared with CE, possibly because of the protective effect of chitosan nanoparticles on the encapsulated CE, which fought against the gastric acid in the stomach and enabled gradual release and absorption of CE in the intestinal tract. The encapsulated CE led to a significant increase in grip strength. However, neither CECNs nor CE could fully restore grip strength to the level of the healthy control group. After the 42-day treatment period, 10% and 20% declines in grip strength were still observed in the CECNs-treated group and CE-treated group, respectively.

The area of the perimysium and endomysium significantly decreased in the STZ-induced group treated with CE and CECNs compared with the normal group (Figure 5c). This decrease resulted in a reduction in grip strength. Moreover, after continuous treatment with CE and CECNs, the area of the perimysium and endomysium increased and recovered, and grip strength increased thereafter.

### 3.7. IHC for IL-6

**Figure 8:**
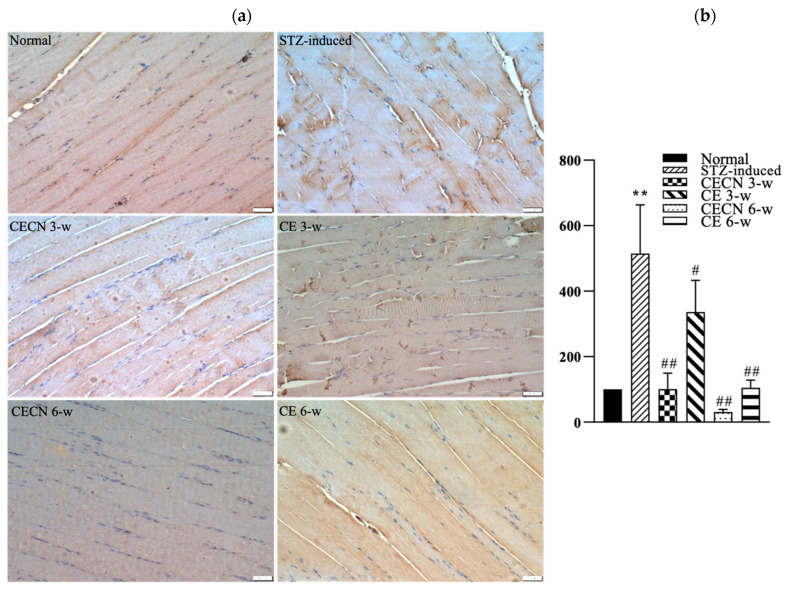
IHC stain and semiquantitative analysis (**a**) IHC for IL-6 in skeletal muscle after 3 and 6 weeks of treatment. (**b**) Semiquantitative analysis of IL-6 staining between groups (n = 4 images) ** *p* < 0.01 as compared with the normal group. # *p* < 0.05, ## *p* < 0.01 as compared with the STZ-induced group. Bar scale: 50 μm.

We next used IHC to detect inflammatory factor IL-6 production after treatment. A less intense brown color, indicating the presence of lower levels of IL-6, observed in the muscle treated with CECN for 6 w or CE for 6 w than in the STZ-induced muscle treated with CE for 3 w (Figure 8a). The muscle treated with CECN for 6 w displayed the lowest staining of IL-6, with more even distribution and normal striated appearance of muscle fibers throughout than the muscle treated with other groups. By contrast, higher IL-6 production with disturbed muscle fibers were noted in the STZ-induced groups treated with CE for 3 w. Among these, treatment with CECN for 6 w is more effective than other groups. As illustrated in Figure 8b, we semiquantitatively determined IHC IL-6 staining intensity after 3 and 6 weeks of treatment. The STZ-induced group exhibited highest inflammation. The inflammation reaction was alleviated after treatments with CECNs or CE and CECN for 6 w exhibited lowest IL-6 staining intensity, indicating that CECN for 6 w exerted an obvious anti-inflammatory activity on the muscle. The results for IL-6 staining demonstrate that CECNs and CE have a considerable capability to reduce the inflammation response in STZ-induced muscle. However, a longer period of 6 weeks of treatment is needed to acquire the anti-inflammation response to repair the STZ-induced muscle.

**Figure 9:**
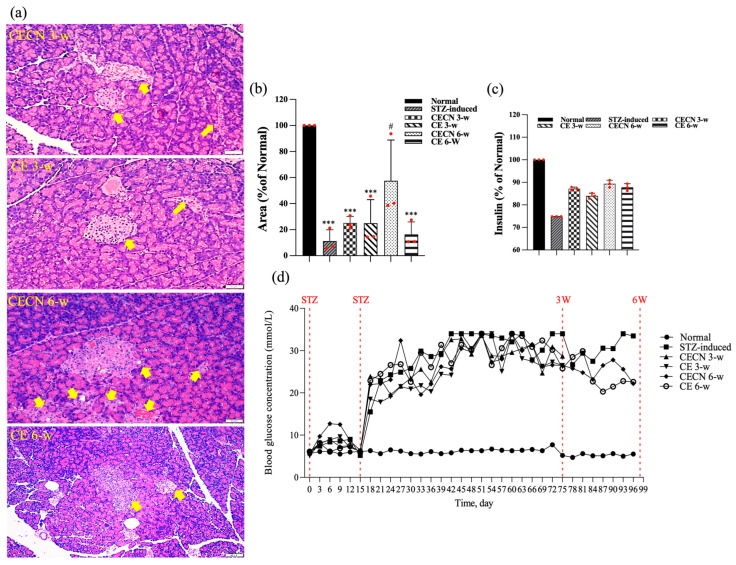
(**a**) H&E stain. (**b**) Semiquantitative determination of pancreatic islets of Langerhans area (n = 3 images). (**c**) Assay of insulin levels in the pancreas at the end of experiment. (**d**) Blood glucose levels throughout the animal experiment. The yellow arrow indicates pancreatic islets of Langerhans. *** *p* < 0.0001 compared with the normal group; # *p* < 0.05 compared with the STZ-induced group. Bar scale: 50 μm.

At the end of the experiment, this study also examined the effects of long-term treatment of the pancreas with CE and CECNs. The histopathological analysis of the pancreas (Figure 9a) revealed that treatment with CECNs for a 6-week period resulted in the restoration of the histopathological phenotype and considerable increases in the number and size of pancreatic islets of Langerhans as compared with STZ-induced groups (Figure 9b). Furthermore, fatty liver was slightly ameliorated after treated with CE and CECNs (images not shown). However, further repair and recovery may still be required to restore the pancreas to a healthy state. Figure 9c presents the insulin secretion levels at the end of experiment; slight increases in insulin levels were observed after treatment with CE and CECNs. Similarly, after 6 weeks of treatment with CECNs, insulin secretion had improved; this finding is consistent with the histological observation of the total area of pancreatic islets of Langerhans in the pancreas. Figure 9d, which presents the blood glucose levels throughout the animal experiment, illustrates that blood glucose levels decreased, albeit not significantly, after treatment with CE or CECNs. Taken together, the present results related to the pancreas indicate that treatment with CE and CECNs can reduce damage to the pancreas by increasing the number and size of β cells, by promoting insulin secretion, and by lowering blood glucose levels in STZ-induced diabetic rats.

## 4. Discussion

The presence of elevated extracellular matrix levels in diabetic tissue samples has been reported in a number of studies. Abnormal extracellular matrix (ECM) turnover can interfere with growth factor signaling and thus can impede myogenesis. In addition, remodeling of the ECM in diabetic mice is advised on the basis of evidence of increased collagen presence, which affects satellite cell functionality and migration, both of which in turn influence muscle growth, maintenance, and repair [28,29,30]. Consistent with the findings of previous studies, in the present study, increased collagen deposition was observed between the muscle fibers of STZ-induced diabetic rats, as was the aggregation of muscle cells and inflammatory cells located between muscle. These changes are associated with muscle atrophy and a decrease in grip strength, as evidenced by the narrowed perimysium and endomysium space between muscle fibers in this study. Importantly, after treatment with CE and CECNs, both the area of the perimysium and endomysium between muscle fibers and the area of muscle fibers (size) had significantly increased, indicating the amelioration of muscle atrophy. Furthermore, the significant recovery of grip strength to more than 80% of that of the healthy control rats indicated the partial recovery of muscle function (Figure 7). However, interpreting muscle biopsies can be challenging, because morphological changes alone may not be sufficient to assign a specific diagnosis; diagnosing muscle changes or diseases often requires a combination of multiple changes observed in a biopsy.

Prolonged exposure to inflammation creates a state of chronic inflammation which involve complicated processes of cytokine release and the emergence of a high-oxidative-stress status, which can cause irreversible cellular and tissue damage. In turn, excessive oxidative stress caused by reactive oxygen species (ROS) can trigger an inflammatory response to lead to further oxidative stress and chronic inflammation. Studies indicated that persistent inflammation caused extreme pro-inflammatory cytokines production and linked chronic inflammation to such as type 2 diabetes and arthritis diseases [31]. Atrophy is a condition that affects skeletal muscle and is characterized by the loss of muscle mass and muscle function. The mechanisms underlying muscle atrophy are complex and multifaceted. TNF-α is the dysregulation of inflammatory cytokines playing a crucial role behind muscle atrophy. IL-6 is also a classic pro-inflammatory cytokine, which possess ancillary function of influencing metabolism. In a rodent study, IL-6 was reported to induce atrophy via downregulation of ribosomal S6 kinase phosphorylation [32,33]. This study revealed that CE and CECNs was successful in decreasing the pro-inflammatory cytokines IL-6, TNF-α and IL-1β levels in in vitro cellular tests (Figure 4c,d). Furthermore, treatment with CE and CECNs decreased the IL-6 level to ameliorate skeletal muscle atrophy and, significantly improved the grip strength in STZ-induced diabetic rats (Figure 7).

Streptozotocin (STZ) is a naturally occurring compound that specifically targets insulin-containing β cells, causing pancreatic β-cell destruction and is widely used to produce an animal model of diabetic mellitus. In this present study, we utilized a multiple, low-dose STZ approach only partially damaged pancreatic islets, triggering inflammatory responses that caused gradual loss of β-cell activity that resulted in insulin deficiency and hyperglycaemia. This response more closely resembled type I diabetes in pathogenesis and morphologic changes than the single, high-dose STZ protocol. In addition, different sensitivity to STZ-induced diabetes has been reported in male and female mice [34]. Kim et al. indicated that fasting blood glucose was about 50% higher in C57BL/6J male mice than female mice at 5 weeks after STZ injection [35]. Another study in C57BL/6 mice reported similar results, i.e., blood glucose was about 90% higher in male mice than female mice [36]. These data indicated that male mice were more sensitive to STZ and tended to develop greater hyperglycaemia. In other words, male mice were more susceptible to STZ to induce diabetes. Indeed, both sexes should be studied to elucidate the determinants of these fundamental biological sex differences from genes to hormones. Further, the identification of sex differences in metabolic function and disease would provide knowledge to allow the development of relevant sex-based therapeutic avenues for diabetes. When considering the efficacy of STZ-induction to diabetes, we utilized male rat as animal target in the present study. As shown in Figure 9, the abnormal pathological conditions originated in STZ-induced diabetic rats, such as shrank pancreatic islet and decreases in the number and size of pancreatic islet in the pancreas, were ameliorated by oral administration of CE and CECNs. Furthermore, serum insulin secretion was slightly increased, and blood glucose levels were lowered under the action of increased insulin levels. These findings indicated that CE and CECNs exerted a reparative effect on the pancreas in the STZ-induced diabetic rats.

**Figure 10:**
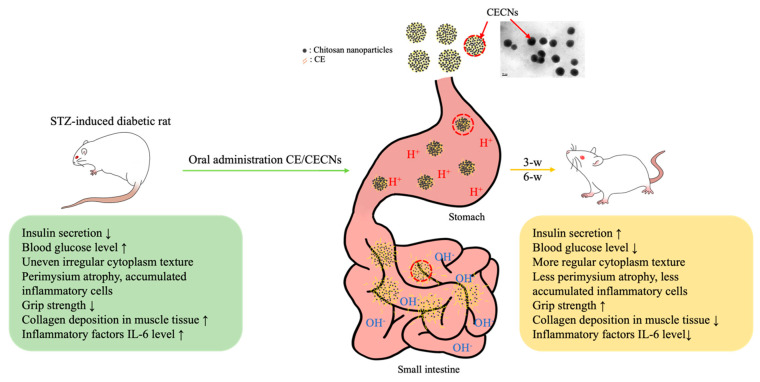
Schematic representation of the probable release of encapsulated CE from CECNs through digestive tract. CECNs treatment led to a reduction of damage to the pancreas, restore normal morphological phenotype and ameliorate muscle atrophy in STZ-induced rats.

In our previous studies, we have successfully produced chitosan and hyaluronan nanoparticles to encapsulate or aggregate poorly soluble drugs in order to enhance the therapeutic efficacy of these drugs [37]. In particular, the positive [H^+^] charge of chitosan nanoparticles, generated in an acid solution environment prepared using NaOH gelation agent during the preparation process, plays a crucial role in protecting the encapsulated drug from gastric acid degradation during oral administration. Aggregated chitosan nanoparticles have the ability to disintegrate in the alkaline environment of the intestine, thereby facilitating the controlled release of the encapsulated drug (Figure 10). On the basis of these unique characteristics of chitosan nanoparticles, CECNs were observed to exhibit a more marked therapeutic effect than CE alone. This effect was confirmed on the basis of histological analysis, significant changes in biochemical test results and improvements in muscle grip strength.

This study revealed many novel aspects of cumin extract effect on restoration of muscle grip strength and protection of pancreas organ in STZ-induced diabetic rats. However, some tests of this research, such as measurement of the anti-oxidative capability of cumin extract, were not performed, as we believed that the antioxidant properties of cumin extract had already been elucidated and proved [38]. This study suggests that the oral administration of cumin extract (CE and CECNs) may be responsible for the lowering of inflammation response, improved muscle grip strength, a reduction of damage to the pancreas, restoring its morphological phenotype, increasing serum insulin levels, and lowering blood glucose levels in STZ-induced diabetic rats. However, further research is suggested in order to obtain the benefits of cumin supplementation in a clinical setup.

## 5. Conclusions

The study demonstrated that cumin aldehyde, extracted through a simple water distillation method involving a rotary evaporator, can be encapsulated in chitosan nanoparticles. In this study, this encapsulation process resulted in lower cytotoxicity and enhanced anti-inflammatory effects given that the cumin aldehyde was slowly released from the nanoparticles. In the present in vivo animal experiments, evidence from histological observations, biochemical tests, and muscle grip strength measurements indicated that treatment with CE and CECNs ameliorated muscle atrophy, increased muscle grip strength, and repaired damaged pancreatic tissue in STZ-induced diabetic rats. These findings suggest that CE and CECNs have the potential to improve muscle function in diabetic rats.

## Data Availability

The data that support the findings of this study are available within this article.
